# Cannabidiol Reduces Leukemic Cell Size – But Is It Important?

**DOI:** 10.3389/fphar.2017.00144

**Published:** 2017-03-24

**Authors:** Nikoletta Kalenderoglou, Tara Macpherson, Karen L. Wright

**Affiliations:** Division of Biomedical and Life Sciences, Faculty of Health and Medicine Lancaster UniversityLancaster, UK

**Keywords:** cannabidiol, Jurkat, cell size, ribosomal protein S6, leukaemia, protein kinase B, physiological normoxia

## Abstract

The anti-cancer effect of the plant-derived cannabinoid, cannabidiol, has been widely demonstrated both *in vivo* and *in vitro*. However, this body of preclinical work has not been translated into clinical use. Key issues around this failure can be related to narrow dose effects, the cell model used and incomplete efficacy. A model of acute lymphoblastic disease, the Jurkat T cell line, has been used extensively to study the cannabinoid system in the immune system and cannabinoid-induced apoptosis. Using these cells, this study sought to investigate the outcome of those remaining viable cells post-treatment with cannabidiol, both in terms of cell size and tracking any subsequent recovery. The phosphorylation status of the mammalian Target of Rapamycin (mTOR) signaling pathway and the downstream target ribosomal protein S6, were measured. The ability of cannabidiol to exert its effect on cell viability was also evaluated in physiological oxygen conditions. Cannabidiol reduced cell viability incompletely, and slowed the cell cycle with fewer cells in the G2/M phase of the cell cycle. Cannabidiol reduced phosphorylation of mTOR, PKB and S6 pathways related to survival and cell size. The remaining population of viable cells that were cultured in nutrient rich conditions post-treatment were able to proliferate, but did not recover to control cell numbers. However, the proportion of viable cells that were gated as small, increased in response to cannabidiol and normally sized cells decreased. This proportion of small cells persisted in the recovery period and did not return to basal levels. Finally, cells grown in 12% oxygen (physiological normoxia) were more resistant to cannabidiol. In conclusion, these results indicate that cannabidiol causes a reduction in cell size, which persists post-treatment. However, resistance to cannabidiol under physiological normoxia for these cells would imply that cannabidiol may not be useful in the clinic as an anti-leukemic agent.

## Introduction

The anti-cancer potential of the phytocannabinoids has been of great interest for the past couple of decades. The non-psychoactive nature of cannabidiol (CBD), has prompted many studies, both *in vivo* and *in vitro*, that support a role for CBD in tumor regression and inhibition of cell viability through reactive oxygen species (ROS)-driven and caspase-dependent apoptosis (reviewed by McAllister et al., 2015). However, the anti-cancer potential of CBD has not been translated into the clinic. A few reasons for this have been asserted, such as incomplete growth inhibition and less than optimal assay conditions (oxygen levels, presence of serum, monocultures) (Fowler, 2015). In addition, the narrow therapeutic window for CBD might be prohibitive (Massi et al., 2013) and, although multidrug therapy could be a solution, drug interactions and metabolism would require further careful analysis. However, one study showed a dose-dependent management of acute lymphoblastic leukemia in one patient with oral cannabinoid extracts (Singh and Bali, 2013). The dose of CBD in this study was not clearly defined.

The Jurkat leukaemic T-cell line is one of the best known model systems for T-cell receptor (TCR) signaling and T-cell acute lymphoblastic leukemia (ALL) since they were purified from a 14 year old patient (Schneider et al., 1977). They have also been used extensively to explore the role of cannabinoids in the immune system, particularly since expression of the cannabinoid receptor-1 (CB1) is absent in these cells (Bouaboula et al., 1993) and can be upregulated through activation (Daaka et al., 1996) and by the phytocannabinoid Δ^9^-tetrahydrocannabinol (THC) (Jia et al., 2006; Börner et al., 2007). This absence subsequently revealed an important role for the CB2 receptor in apoptosis of malignant lymphoblasts (McKallip et al., 2002). However, aberrant CB2 receptor signaling in Jurkats, both in terms of adenylate cyclase inhibition (Schatz et al., 1997) and intracellular calcium mobilization (Rao et al., 2004), has rendered these cells useful for identifying non-cannabinoid receptor effects. Indeed, this was shown for the endogenous cannabinoid, anandamide (AEA), such that the induced apoptosis in Jurkats was via a membrane lipid raft mechanism and not via vanilloid (TRPV)-1 or CB1/2 receptors (Sarker and Maruyama, 2003). This was similarly true for the CB1/VR1 hybrid molecule, arvanil, in that induced apoptosis was non-receptor mediated and largely via the intrinsic pathway with some involvement of the plasma membrane NADH-oxido-reductase system (Sancho et al., 2003).

Subsequent studies using this cell line and THC, established the intrinsic pathway of apoptosis as being critical (Lombard et al., 2005), but, importantly, that the CB2 receptor did in fact mediate this effect through an increase in p38 MAPK activity (Herrera et al., 2005) and ceramide production (Herrera et al., 2006) and/or through a reduction in the Raf-1/MEK/ERK signaling pathway (Jia et al., 2006). With respect to CBD, a meticulous study of CBD-induced apoptosis revealed a dependence on the CB2 receptor for the expression of the NAD(P)H oxidases p22*^phox^* and Nox4, ROS production and caspase activation (McKallip et al., 2006).

In this study, we sought to explore the issue of incomplete cell death in Jurkat T cells in response to CBD. Inhibition of survival and cell growth pathways by cannabidiol revealed a potential role for the ribosomal S6 protein, which has been reported to be a key regulator of cell size independent of its role in protein translation (Ruvinsky et al., 2005). We then questioned whether CBD had an impact on cell size and, if so, whether it was reversible. To strengthen the translatability of these findings, we also checked whether physiological normoxia altered the cannabidiol effect on cell viability.

## Materials and Methods

### Drugs and Reagents

Cannabidiol was obtained from Tocris Bioscience (Bristol, UK). CBD was initially dissolved in DMSO (stock concentration of 75 mM) with subsequent dilutions in serum-free tissue culture medium for *in vitro* experiments. Doxorubicin hydrochloride was purchased from Abcam (Cambridge, UK) and the stock dilution in DMSO to 25 mM was diluted in serum-free medium. The following antibodies were purchased from Cell Signaling Technology [New England Biolabs (NEB), Hertfordshire, UK]: PathScan^®^ Multiplex Western Cocktail I (#5301), anti-Phospho-mTOR (Ser2448) (D9C2) XP^®^ Rabbit mAb (#5536), β-Actin (8H10D10) Mouse mAb (#3700), anti-Rabbit IgG, HRP-linked Antibody (#7074) and anti-Mouse IgG, HRP-linked Antibody (#7076). Concanavalin A was from Sigma–Aldrich, UK (#C2272) with stock solutions stored at 5 mg/mL in dH_2_O.

### Cells

The human leukaemic cell line Jurkat were maintained in RPMI 1640 medium supplemented with 10% fetal bovine serum (complete medium defined as nutrient rich conditions) (both from Invitrogen, Paisley, UK). For routine culture, medium was changed every 2–3 days and cell density was maintained between 5 × 10^5^ and 2 × 10^6^ cells/mL. Standard incubator conditions were 21% O_2_, 5% CO_2_, and 37°C, denoted AtmosO_2_. Serum-free medium (defined as nutrient poor conditions) was used in signaling experiments and to initiate the CBD effect in recovery experiments, with complete medium used in the recovery phase. Cells were also cultured in physiological normoxia (12% O_2_ and 5% CO_2_, denoted PhysO_2_) in an H35 Hypoxystation from Don Whitley Scientific, Shipley, UK. Normal cell culture used 5% serum-containing RPMI. For activation experiments, 10^6^ cells/mL from both AtmosO_2_ and PhysO_2_ were washed once with RPMI and cultured in normal growth medium supplemented with 5 μg/mL Concanavalin A for 48 h.

### Viability Assays

Exponentially proliferating cells were counted and 10^5^ cells/mL were seeded into 96-well plates in medium containing 10, 5 or 1% serum, as indicated. Cells were incubated for 72 h with or without compounds, as indicated. After the time period, 20 μL of PrestoBlue^®^ reagent (Invitrogen, Paisley, UK) was added to wells and cells further incubated for 2 h. Changes in fluorescence were measured at 560 and 590 nm. For doxorubicin experiments, cells were incubated for 72 h in 5% serum conditions in both AtmosO_2_ and PhysO_2_. All experimental values were determined from triplicate or quadruplicate wells. After subtracting the average fluorescence values of the no-cell control wells from all the experimental wells, the data was averaged and depicted as a percentage of untreated control.

### Determination of Cell Size

Viable cells exclude trypan blue, whereas dead and dying/apoptotic cells are able to take up the dye since their membranes are compromised. Normally cultured viable Jurkat cells are between 10 and 14 microns (Rosenbluth et al., 2006). Using an automated cell counter TC20 (Bio-Rad), small cells were regarded as those within 5–9 microns and only those that excluded trypan blue were counted. Cells greater than 14 microns were also present, but in negligible numbers, that did not change. Giant cells greater than 18 microns were rare in normal culture. Viability experiments were carried out in 6-well plates and starting cell densities were always 10^6^ cells/mL. After treatments and times, as indicated, cells were resuspended and aliquots were counted in 1:1 trypan blue (0.4%) solutions within 1–2 min to avoid toxicity. Whole cell counts were recorded as live cells/mL and percentage viability, with subsequent gating for live small cell counts (S) and live normal size cell counts (N), based on the above parameters. These counts were expressed as a percentage of the total live cell count or as cells/mL as indicated. In addition, cell aliquots stained with 1:1 trypan blue (0.4%) were placed on microscope slides and coverslips were applied. Phase contrast images of the slides were acquired using a VisiCam TC10 tablet (VWR International, Leicestershire, UK) fitted onto a Motic BA210 Upright Microscope (Motic, Hong Kong) with a 20× objective lens.

### Recovery Experiments

Proliferating cells from culture were resuspended in serum-free RPMI medium at 10^6^ cells/mL and treated with or without CBD at 10 μM for 24 h. Cells were counted and resuspended in complete medium (10^6^ cells/mL) without CBD for a further 24 h. This was repeated for two more rounds up to 96 h (72 h post-treatment). In other experiments, cells were allowed to recover for 24 h and then CBD treated again for 24 h, with a subsequent 24 h recovery.

### SDS-PAGE and Immunoblotting

Cells from experiments (adjusted to 10^6^ cells) were lysed at the times indicated in 100 μL of Pierce^TM^ IP Lysis Buffer (#87787) with Halt^TM^ Protease Inhibitor Cocktail (#78430), both from Thermo Scientific, UK. Lysates were boiled under reducing conditions in 4X Laemmli sample buffer (Bio-Rad, Hertfordshire, UK). Sample proteins were separated using precast 4–16% TGX Stain-Free gels and transferred onto nitrocellulose membrane (Bio-Rad, Hertfordshire, UK) before blocking with 5% bovine serum albumin (BSA) in Tris-buffered saline with 0.1% Tween (TBST) at RT for 1h. Primary antibodies were incubated overnight at 4°C as per manufacturer’s instructions and, after washing membranes in TBST, blots were incubated with the appropriate secondary antibody at RT for 1 h. Blots were exposed using Clarity^TM^ Western ECL Substrate and imaged with the Bio-Rad ChemiDoc^TM^ XRS system. Quantitation of blots was derived from the imaging system from which the relative amount of protein to Rab11 was calculated and used for normalization.

### Flow Cytometry

Distribution of cells in the cell cycle was determined by propidium iodide (PI) staining and flow cytometry analysis using a FACS Canto II (BD Biosciences, Oxford, UK). Briefly, 10^6^ cells from experiments were fixed in 90% ethanol, washed and resuspended in RNase A (10 μg/mL) and PI (50 μg/mL). Ten thousand events were collected and live cells were gated away from dead cells as well as debris based on the FSC/SSC parameters. This cell population gate is then placed on PE-Width versus PE-Area dot plot to exclude clumped cells from single cells. The mean FSC-H of the single cells was determined as a measure of relative cell size. Data from the flow cytometry measurements were analyzed using the CytExpert software (Beckman Coulter, Buckinghamshire, UK).

### Statistical Analysis

Data are expressed as the mean ± SD of *n* experiments. To determine statistical significance, Student’s *t*-test was used for comparing a single treatment mean with a control mean, and a one-way ANOVA followed by a Dunnett’s multiple comparisons test was used for analysis of multiple treatment means. *P*-values < 0.05 were considered significant.

## Results

### Effect of Cannabidiol on viability and Cell Cycle in Jurkat Cells

**FIGURE 1:**
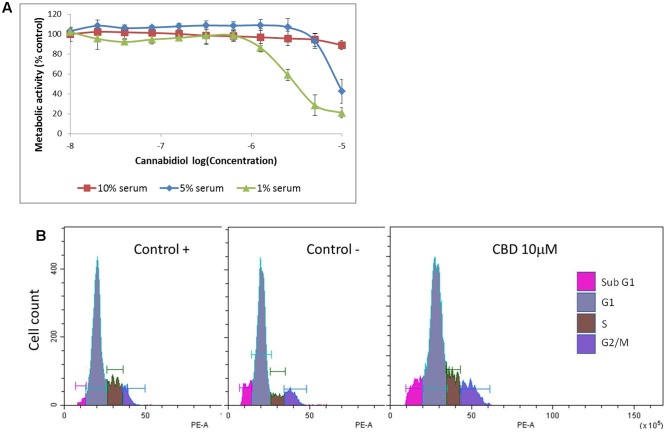
**Cannabidiol affects Jurkat cell viability and cell cycle progression.** **(A)** Cells were treated with CBD (0–10^-5^ M) for 72 h in RPMI with 10% serum (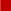), 5% serum (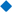), and 1% serum (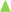). Cell viability was measured using the PrestoBlue^®^ assay. Results are expressed as average percentage viability (±SD) relative to untreated controls, *n* = 3. **(B)** Cell cycle histograms obtained by population-based DNA content analysis using flow cytometry, see **Table 1**.

Previous studies have indicated that CBD effects are influenced by the presence of serum, and previous data on Jurkat cells were generated in serum-free conditions (McKallip et al., 2006). To vous findings, Jurkat cells were exposed to cannabidiol (0.01–10 μM) for 72 h in a range of nutrient conditions (**Figure 1A**). This resulted in a significant inhibitory effect on the viability of cells at concentrations greater than 1 μM in low serum conditions. This effect was dependent on the nutrient conditions since only 10 μM of CBD was able to inhibit cellular respiration in nutrient rich conditions. IC50 values of 6.4 ± 2.9 and 2.5 ± 0.2 μM were obtained for cells kept in 5 and 1% serum, respectively. To check the cell cycle status of treated cells, cells from treatments in serum-free conditions were removed for DNA content analysis using PI (**Table 1** and **Figure 1B**). Cannabidiol delayed the cell cycle when compared to control -, with an increase in G1 cells and a reduction of cells in the S and G2/M phases. The percentage of SubG1 cells (late apoptosis) remained the same.

**Table 1:** Cell cycle distribution.

Cell cycle stage	Control +	Control -	CBD (10 μM)
Sub G1	2.18 ± 0.2	5.63 ± 0.6	5.14 ± 0.7
G1	70.94 ± 0.9	66.93 ± 0.9	78.21 ± 1.7^∗^
S	18.99 ± 0.6	15.01 ± 1.5	8.9 ± 1.5^∗^
G2/M	7.4 ± 1.4	12.11 ± 1.4	8.74 ± 0.8^∗^


*Cells were cultured in complete medium (control +), serum-free medium with CBD (10^-5^ M) or without (control -) for 24 h. The percentage (± SD) of cells in each phase of the cell cycle is shown, (^∗^*p* < 0.05) *n* = 4.*

### Cannabidiol Deactivates the mTOR Pathway

**FIGURE 2:**
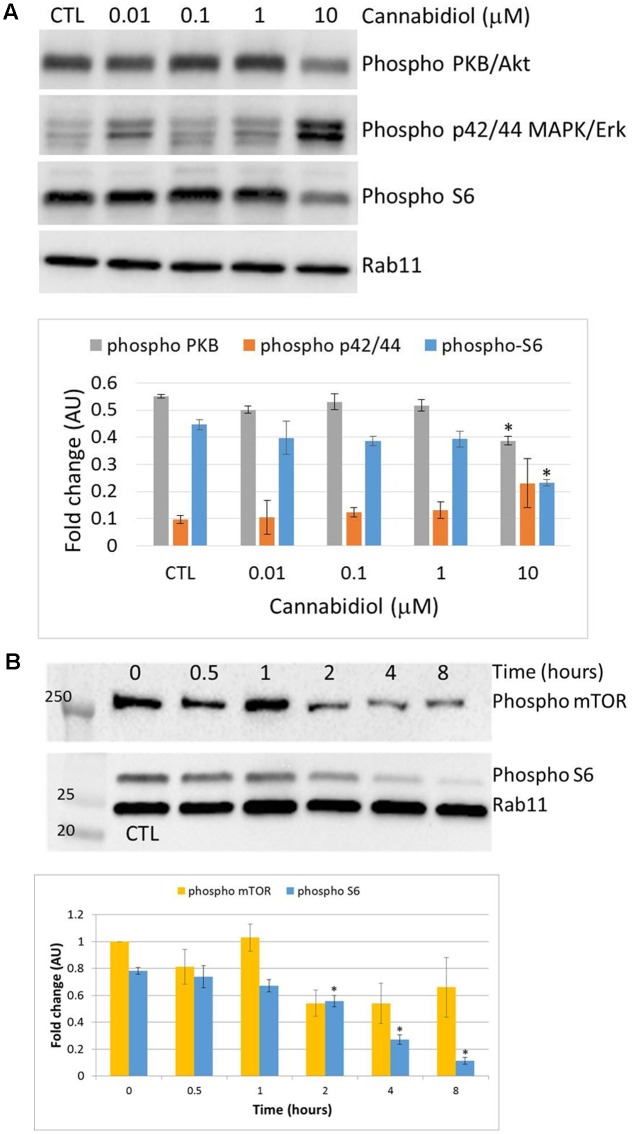
**Cannabidiol-induced mTOR and S6 dephosphorylation.** **(A)** Representative blots of CBD dose response (10^-5^–10^-8^ M) on PKB, p42/44, and S6 phosphorylation, *top panel*. CTL denotes cells treated with vehicle alone. **(B)** Time course up to 8 h of S6 and mTOR phosphorylation with and without CBD (10^-5^ M), *top panel. Bottom panels* show the intensity of protein bands normalized to Rab11, calculated as fold difference from controls (mean ± SEM), *n* = 3 (^∗^*p* < 0.05).

Cannabidiol has previously been shown to deactivate Protein Kinase B/Akt (PKB) and mammalian Target of Rapamycin (mTOR) in breast cancer cells (Shrivastava et al., 2011), so we performed a dose response over 4 h to confirm this in Jurkat cells (**Figure 2A**). CBD reduced the phosphorylation levels of PKB and the ribosomal protein S6 only at 10 μM. There did appear to be an increase in the p42/44 MAPKs but this was not significant. We then performed a time course with CBD for up to 8 h (**Figure 2B**). For the time course, we included the phosphorylation of mTOR to correlate with the S6 response. CBD did deactivate mTOR to some extent, but this was not significant with multiple comparisons. However, the deactivation of S6 was time-dependent, reaching significance by 2 h.

### Recovery of Cannabidiol-Treated Cells

**FIGURE 3:**
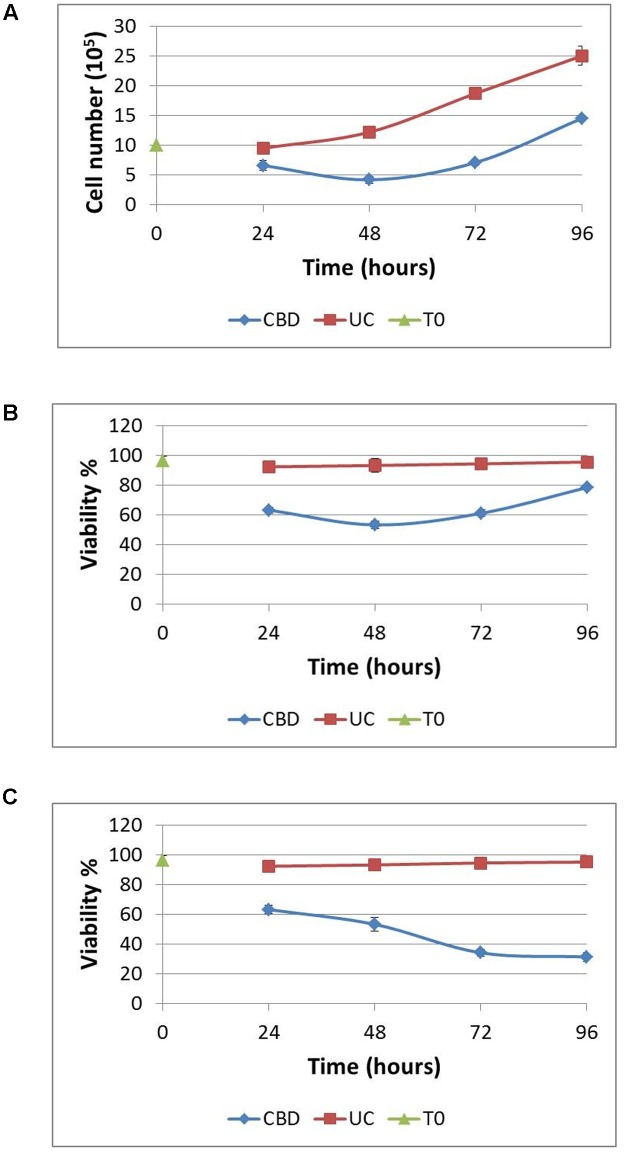
**Recovery from cannabidiol treatment.** 10^6^ cells/mL were treated with or without CBD (10^-5^ M) for 24 h in serum-free medium, resuspended in complete medium without CBD at a density of 10^6^ cells/mL for 24 h. This was repeated at 48 h for a further 24 h. Cells were counted daily **(A)** and viability calculated **(B)**, UC denotes untreated control. In one set of experiments, CBD (10^-5^ M) was reapplied at 48 h in serum-free medium and allowed to recover for 24 h in complete medium without CBD **(C)**. Viability is expressed as the percentage of live cells in a total cell count, *n* = 3.

Next, we sought to ascertain whether cells that remain viable after CBD treatment for 24 h in serum-free conditions, could recover their proliferative capacity. Complete medium was replaced daily after the CBD treatment to a density of 10^6^ cells/mL. Using the Trypan Blue exclusion principle, cells were counted at the start and daily throughout the experiment (**Figure 3A**). Overall, viable cell numbers were reduced by 30% when CBD was applied at 10 μM. This effect persisted into the next day, despite complete medium replacement, with cell numbers returning to their starting level after 72 h recovery. However, this recovery did not reach the same levels as untreated cells, which more than doubled in number. At the same time, we analyzed viability, based on live cells as a percentage of total cell count (**Figure 3B**). This reflects the cell count analysis; in that cells did not entirely recover basal viability in this time frame. To try and mimic a multiple dosing regime, cells that were allowed to recover for 24 h and then again treated with CBD in serum-free conditions, had a further reduction in viability to 34.2 ± 1.4 %, which did not recover (**Figure 3C**).

### Cannabidiol Reduces Cell Size

**FIGURE 4:**
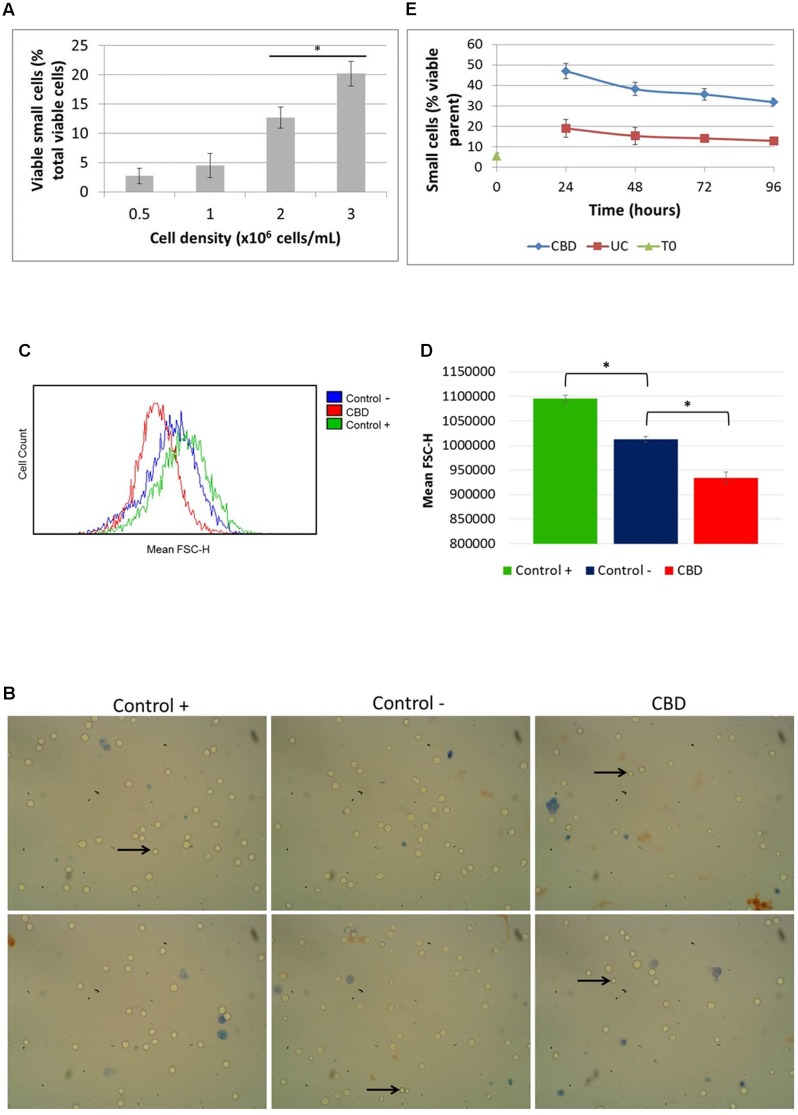
**Cell size distribution.** Cells in routine culture at different densities were counted and size-gated **(A)**. Cells in complete medium (control +), or serum-free medium in the absence (control –) or presence of CBD, were incubated for 24 h **(B–D)**. **(B)** Representative phase contrast microscope images of cells stained with Trypan Blue, with arrows indicating small viable cells as seen by eye. Mean FSC-H histograms **(C)** with quantitation **(D)**, (^∗^*p* < 0.05) *n* = 4. **(E)** Cells from recovery experiments (see **Figure 3B**) were counted and sized daily. Data is depicted as the percentage of viable small cells in the viable parent population, (^∗^*p* < 0.05) *n* = 3.

Since we noted the significant reduction in phosphorylation of S6 by CBD and had observed smaller cells in normal cell counts visualized under the microscope (**Figure 4B**, *left panel*), we checked the actual cell size change using automated multifocal plane analysis of cells gated for size. Gating was based on previous evidence (Rosenbluth et al., 2006) and we found that in cycling asynchronous cultures, the percentage of so-called small cells increases as cell density increases (**Figure 4A**). It should be emphasized here that these small cells are not dead or dying cells; they exclude Trypan Blue and are usually counted as viable in total cells counts (which takes into account all viable cells, regardless of size). Treated cells were also imaged (**Figure 4B**) and analyzed using flow cytometry (**Figures 4C,D**). Cells were cultured in nutrient rich medium (10% serum, control +), serum-free medium (control -) and with CBD (10 μM) in serum-free medium for 24 h (**Figures 4B–D**). The images in **Figure 4A** show the small cells seen by eye. However, the mean FSC-H, which is a measure of relative cell size based on the granularity of cells, was found to be significantly less in cells in serum-free medium compared to normal culture conditions (**Figure 4D**). More importantly, CBD further reduced the mean FSC-H, seen as a left-shift in the histogram in **Figure 4C** and quantified in **Figure 4D**. Performing the recovery experiment (see **Figure 3B**) and automated multifocal plane analysis, we found that CBD significantly increased the percentage of small cells over that of control (**Figure 4E**) and that despite medium replenishment, this population ratio persisted.

### Physiological Normoxia Renders Cells Resistant to CBD

**FIGURE 5:**
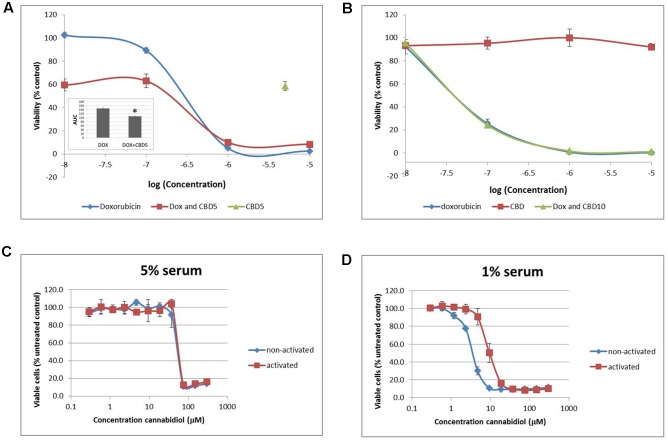
**Physiological normoxia impacts on Jurkat cell sensitivity to cannabidiol.** Cells from either AtmosO_2_ **(A)** or PhysO_2_ **(B)** cultures were seeded into 96-well plates (10^5^ cells/well) in medium (with 5% serum) with or without CBD (5 or 10 μM, indicated as CBD5 or CBD10) and/or DOX (10^-5^–10^-8^ M) for 72 h. Cell viability was measured using the PrestoBlue^®^ assay. Results are expressed as average percentage viability (±SD) relative to untreated controls. Area under the curve (AUC) analysis (inset in **A**) (^∗^*p* < 0.05), *n* = 3. An increased dose response was performed with CBD up to 300 μM on both activated and non-activated cells from PhysO_2_ conditions in medium with 5% serum **(C)** or 1% serum **(D)**, *n* = 4.

To try and replicate these results and potentially explore further mechanisms for our results, we sought to improve our cell model to more closely reflect model the *in vivo* conditions that Jurkat T cells originated from. Cells were transferred and cultured in complete medium in an environment-controlled incubator at 12% O_2_ (termed PhysO_2_). Whilst T cells can be found in a wide range of oxygen environments in the body, arterial blood is at 12% (Atkuri et al., 2007). Doxorubicin (Dox) is a drug used to treat many types of cancer and is approved for use in ALL. It is an anthracycline antibiotic and has previously been shown to induce apoptosis in Jurkat cells (Finlay et al., 1986; Mendivil-Perez et al., 2015). In our hands, Dox reduced cell viability with an IC50 = 200.8 ± 24.4 nM (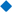) in standard culture conditions (AtmosO_2_) (**Figure 5A**). In combination (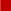) with a sub-lethal dose of CBD (5 μM) (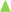), there was a significant reduction in the AUC by approximately a third (**Figure 5A**, inset). However, this was only an additive effect at low concentrations of Dox that did nothing alone (10^-7^–10^-8^ M), and the level of reduced viability seen in this range was the same as CBD alone (∼40% reduction). Next, performing the dose responses to Dox and CBD in PhysO_2_ conditions (**Figure 5B**), revealed a resistance to CBD alone (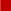), and an increased susceptibility to Dox (IC50 = 46.7 ± 6.7 nM) (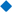). Compare this with an IC50 of 200 nM seen in AtmosO_2_ conditions. In addition, there was no additive or synergistic effect of CBD in combination with Dox (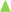).

We found this surprising and sought to verify the lack of response to CBD by performing a dose response up to 300 μM. In 5% serum conditions, Jurkat cells did respond to CBD, but only at concentrations greater than 37.5 μM with an IC50 = 43.4 ± 5.1 μM (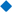) (**Figure 5C**). Since Jurkat cells are T cells and can be activated by mitogen (Daaka et al., 1996), we wondered whether this phenotype would respond to CBD. Activated cells responded to CBD in the same way as non-activated cells in the AtmosO_2_ environment (data not shown) but in the PhysO_2_ environment, these cells are also resistant to CBD effects up to 37.5 μM (**Figure 5C**) (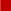). Interestingly, the interaction effect between the activated and non-activated cells are statistically significant, with activated cells more resistant to CBD-induced cell death (IC50 = 63.4 μM). Our earlier result (**Figure 1A**) showed that 1% serum conditions produced a robust dose-dependent CBD-induced reduction in cell viability over 72 h and the serum-free effects of CBD on Jurkats was also previously shown by McKallip et al. (2006). We then repeated these experiments in 1% serum conditions with both activated and non-activated cells cultured in the PhysO_2_ environment (**Figure 5D**). Under these conditions, there are three significant features. The first is that CBD does reduce cell viability in both cell phenotypes in a dose-dependent manner. Second, the low serum conditions make a difference to the CBD-induced response for both phenotypes and the IC50 for non-activated PhysO_2_ cells (3.2 μM) compares favorably with the AtmosO_2_ cells in **Figure 1A** (2.5 μM). Third, the interaction effect is highly significant, in that activated cells in PhysO_2_ conditions are significantly more resistant to CBD than non-activated cells (IC50 = 10.9 μM).

## Discussion

In this study, we found that CBD induced a reduction in cell viability, both in terms of a reduction in mitochondrial respiration and in overall cell number. However, of the remaining viable cell population, there was an increase in the percentage of small cells, which was not reversible on the return of favorable nutrient conditions. In addition, we found that Jurkat cells cultured in physiological conditions (PhysO_2_) were resistant to CBD alone up to 40 μM, but did respond to the chemotherapeutic agent doxorubicin. Finally, we found that low serum did reveal a dose-dependent effect on both activated cells and non-activated cells in the PhysO_2_ environment, with activated cells more resistant to CBD.

The low efficacy of CBD in serum was first noticed when the antibacterial activity seen *in vitro* was reduced 10-fold in serum (Van Klingeren and Ten Ham, 1976). It is thought to be a result of binding to albumin (Papa et al., 1990), but how this affects CBD binding at the membrane is complex. The effect could also be related to cellular stress and different mitochondrial activity under the different nutrient conditions (Jacobsson et al., 2000; McKallip et al., 2002, 2006).

The possibility that anti-cancer medicines might be less effective under nutrient rich conditions is not new and interest in low nutrient stress conditions for chemotherapeutic drug delivery is currently in clinical trial (Lee et al., 2012; Dorff et al., 2016). Our observation that CBD becomes considerably more effective at killing cancer cells under these conditions *in vitro* supports this view. However, recovery experiments in which cells that remained viable after CBD treatment in serum-free conditions began to recover their numbers after a few days, would suggest that incomplete cell death might carry a risk of re-emergence of the leukemia. Repeated doses could have potential, although we did not explore this further in the AtmosO_2_ environment since we wanted to improve on this model.

The reduction in viability by CBD in serum-free conditions had a significant impact on cell size, as measured by flow cytometry and multifocal plane analysis. The reduction in cell size by CBD did not appear to be reversible and the percentage of small cells of the total viable population persisted. The shift to a small cell phenotype may be related to hypodiploid cells, which are a subtype of ALL (Holmfeldt et al., 2013) and particularly resistant to chemotherapy. However, we were unable to differentiate between viable hypodiploid cells and those in late apoptosis, and therefore unable to say whether therapeutic use of CBD in ALL would carry this risk.

Phosphorylation of the ribosomal protein S6 has been shown to regulate cell size independently of its role of S6 in translation, which does not require phosphorylation (Ruvinsky et al., 2005). Jurkat T cells have defective lipid phosphatases, Src homology 2 domain containing inositol polyphosphate phosphatase (SHIP) (Freeburn et al., 2002) and phosphatase and tensin homolog deleted on chromosome 10 (PTEN) (Shan et al., 2000), resulting in constitutive phosphorylation of PKB. Despite these defective pathways, CBD was able to deactivate PKB at 10 μM and this would support the loss of viability. The mTOR signaling pathway is quite complex with phosphorylation status of mTORC1 vs. mTORC2 not explored in our study. PKB is upstream of mTORC1 and mTORC2 is upstream of PKB. Our data appeared to show a reduction in mTOR phosphorylation overall, however, the analysis was not significant. However, the significant deactivation of S6 by CBD would suggest the PKB/mTORC1 route. This deactivation effect phenocopies the rapamycin effect on Jurkat cell size (Fumarola et al., 2005). In this study, the prototype inhibitor of the mTOR/p70 S6 kinase pathway, rapamycin, reduced cell mass and size with slowed proliferation and hints at CBD being an mTOR inhibitor.

Clearly, the mechanisms of this process warrant further study. It is possible that some cells in the population do not die in response to CBD, but rather become resistant to the apoptotic signal by reducing their size. These smaller cells might recover proliferative capacity when conditions are favorable, but they may continue to be resistant to apoptosis. However, this may be completely irrelevant if in the complex *in vivo* environment, some cancer cells display CBD resistance anyway. When these cells were transferred to physiological normoxia, they became resistant to CBD, whether activated or not. We have previously shown that colorectal cancer cells become more sensitive to CBD under more appropriate environmental conditions (Macpherson et al., 2014) and, indeed, in this study, Jurkat cells became more susceptible to doxorubicin-induced cytotoxicity, but CBD-induced reduction in cell viability in the PhysO_2_ environment required considerable doses of CBD. Low serum conditions did reveal the CBD effect, but activated cells were still more resistant.

Low physiological oxygen culture is thought to be important for stem cell culture, possibly due to the amplification of genes involved in metabolic processes (Guo et al., 2013). This mechanism makes sense with respect to the action of CBD, in that mitochondrial respiration changes under different oxidative environments could impact on the ability of CBD to generate ROS thought to be involved in CBD-induced cell death (McKallip et al., 2006). To achieve these doses or the preferred nutrient poor conditions *in vivo*, would render CBD impractical as an anti-leukemic medicine.

## Conclusion

The need for better *in vitro* modeling is of great importance in the preclinical phase of drug development, particularly to reduce failures at the clinical trial phase. Our data adds further support to the assertion that CBD may not be very clinically useful in terms of an anti-cancer medicine *per se*, but may have other medicinal value yet to be clinically proven in oncology.

## Author Contributions

KW was responsible for conception and design, analysis and interpretation of data and redaction of the manuscript. NK and KW were responsible for generation, collection, analysis and/or interpretation of the data. TM performed the viability assays. All authors approved the final version of the manuscript.

## Conflict of Interest Statement

The authors declare that the research was conducted in the absence of any commercial or financial relationships that could be construed as a potential conflict of interest.
